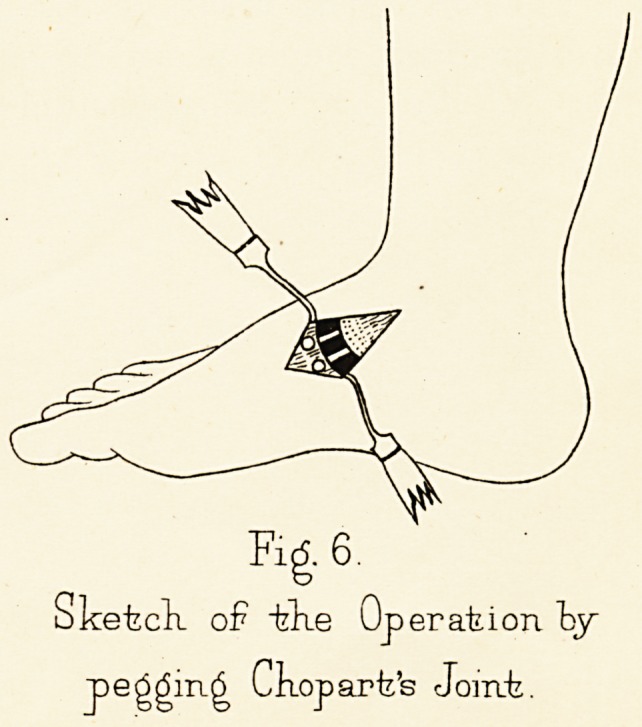# On Flat-Foot and Its Cure by Operation

**Published:** 1884-03

**Authors:** Alexander Ogston

**Affiliations:** Professor of Surgery in the University of Aberdeen


THE BRISTOL

Medico-Chirurgical Journal.

MARCH, 1884.

### ON FLAT-FOOT, AND ITS CURE BY OPERATION.

BY

ALEXANDER OGSTON, C.M.,

*Professor of Surgery in the University of Aberdeen.*

THE improved results of wound treatment by Sir Joseph Lister's antiseptic method have placed in our hands modes of remedying surgical deformities that would formerly have been regarded as unjustifiable, but may now be practised with absolute confidence by skilled antisepticians, and with excellent results even by those less versed in the antiseptic methods.

The operations of osteotomy, though known and practised by a few before Listerism was introduced, have now become the property of every surgeon and are almost universally practised. But much remains to be done before we shall have turned our improved methods of wound treatment to the best possible account in the surgery of bones and joints.

In one part of this field I have been working for several years, and now venture to bring my results before my surgical brethren. The cure of flat-foot is the object I have aimed at.

Excepting those by apparatus, the modes of treating this disease at present employed are, so far as my experience goes, entirely unsatisfactory, and no instance of cure by any of them has come under my observation. It is true that by rest and time the pain that accompanies the deformity becomes ameliorated or disappears, so that the surgeon may, if he pleases, call the result a cure; but the deformity does not disappear or even become materially diminished. A cure, in the proper sense of the word, has not taken place. In all the cases where I have employed the ordinary methods long and patiently there has resulted in the most favourable merely a cessation of the pain at the instep, but in the large majority the patients have wearied of treatment and withdrawn themselves from it. Want of time and money have hitherto prevented such patients carrying out a cure by orthopædic machines under my care.

Flat-foot, or pes valgus acquisitus, is, like scoliosis and knock-knee, almost invariably the result of a disproportion between the strength of the foot and the work it has to accomplish. Hence it generally occurs in adolescents, is most frequently due to the rachitis adolescentium that produces so many of these static deformities, and is seldom found save in those who are overworked. In message boys, young agricultural labourers, domestic servants, and even young people at school, we frequently see it slowly appear. If we examine into their general health we rarely miss the languid circulation, the cold extremities prone to perspire, and the ricketty knottings of the bones, especially at the anterior ends of the ribs. We are told that there has been heavy labour, out of proportion to the strength, causing pain or dull aching about the instep, which passes off on rest but reappears on exertion, and becomes more aggravated the longer the exertion is continued.

But the history of flat-foot varies much. I know of one case where a very marked double flat-foot occurred, without any pain being complained of, in a boy of seven years of age, who was very fat, and in whom the weight of the large body seemed the only cause adequate to account for the onset of the deformity.

In flat-foot, as in all static deformities, it seems reasonable to believe that all the textures which normally contribute to maintain the correct posture of the foot become affected, though of course in different degrees, and the foot, as a consequence, becomes perverted in its form.

The alterations are worthy of some notice. The point on which they hinge is a yielding and flattening of the arch of the instep, all other changes being secondary to this and directly consequent on it. The flattening of the arch of the foot is the test of the existence of flat-foot, and the condition is better designated by the term flat-foot, or pes planus, not unfrequently bestowed upon it, than by the name of pes valgus, or everted foot, that it oftener bears. The name pes valgus is misleading. The valgus or everted posture is not necessarily characteristic, as it includes another and different condition which is, I think, generally confounded with it. This condition is that of everted or valgus ankle.

Valgus ankle is usually seen in young girls passing out of childhood, and is sometimes temporary, sometimes permanent. The ankle, especially when seen from behind, is observed to have lost its straight form and to fall inwards, so that the malleoli approach the middle line and constitute a "knock-ankle." The rubbed condition of the boot may show that the ankles do actually strike one another in walking. If the foot be inspected without its coverings, the valgus ankle, which may be detectable only when the patient stands, is observed to be limited to the region of the ankle-joint, and seems due to a loss of form of the bones, the malleoli and astragalus, perhaps the calcaneo-astragaloid articulation as well. But the foot below the ankle does not participate in the deformity, save in so far as the valgus ankle entails on it an outward deviation. The arch of the instep remains unaltered (Fig. 1).

In true flat-foot, on the contrary, the ankle-joint can hardly be said to participate. The arch of the foot suffers, and the bones, tendons and ligaments that maintain the shape of the instep are so modified that the arch unfolds, its two extremities recede from one another, and its curve finally becomes a straight line, touching the ground along its whole length.

Examination of such a foot may reveal a slight laxity of all its articulations; but there are always great changes at the joint between the scaphoid and the head of the astragalus, forming the inner half of the medio-tarsal or Chopart's articulation. Here the relaxation is very great, so that by acting on this joint alone we can on the one hand rectify the faulty position of the foot, and on the other hand move it into the worst possible degree of the deformity.

It is only when the disease is still recent that we can by manipulation cause the deformity to disappear. In recent cases it may even disappear if the patient sit down and lift up the foot for our inspection. The superincumbent weight being removed, the flattening of the arch disappears, sometimes quickly, sometimes more slowly, so that the foot may be normal in outline, and our attention be called to what really exists only by the patient complaining of a dull aching pain at the instep, sometimes aggravated by firm pressure about the scaphoid bone. In such instances mistakes in diagnosis are frequent. When the patient stands, however, the deformity appears, and if he again sit down and lift up the foot it resumes once more its normal outline.

When we try manually to enlarge the arch which the instep forms, the deformity can be made as evident as when the patient stands, and we can remedy in like fashion the distortion we have produced. When the foot is so manipulated it is only needful to move Chopart's joint, one hand fixing the astragalus and the other grasping the scaphoid. Then the maximum degree of the disease can be caused to appear or disappear, while similar movement at any other joint shows that its share in producing the deformity is very slight. So long as the hands maintain Chopart's joint in its proper position, the flat-foot cannot be rendered evident.

In process of time the deformity becomes permanent, and can no longer be made to disappear. It is in such cases that the details of the deformity can best be studied. We discover that the tarsal bones along the inner side of the foot are ranged in a line parallel with the ground and everywhere in contact with it. The first metatarsal and the internal cuneiform are horizontal in direction; the scaphoid and head of the astragalus behind them form a marked prominence on the sole and inner side of the foot, a callosity covering them on the sole and a bunion-like patch of skin on the inner side of the foot. The articulalation between them, so mobile in the earlier stage, is now fixed and rigid. On the application of great force it can sometimes be caused to yield, so that Chopart's joint may be restored to position, the deformity disappearing, but often the joint remains rigid and refuses to move upwards under any force. This is due to an alteration in the shape of the bones, detectable by palpation and also visible when the joint is opened.

The manner of production of this alteration is as follows. When the arch is flattened the ligaments beneath become elongated, and on the under surface of the joint the bones tend to become separated from one another, while on the upper side the pressure between them is increased. Hence growth is checked, or absorption even takes place, at the upper halves of the articulations of the scaphoid and astragalus, while the lower halves, where the mutual pressure is lessened, show a tendency to separation and increased growth of the separated surfaces. The increased growth takes place mainly in the astragalus, so that the joint is not found gaping below, but the caput tali is changed in shape, becomes somewhat square in form, and presents an abnormal ridge, or projecting angle, dividing its articular facet into two portions, one articulating with the scaphoid, the other with the inferior calcaneo-scaphoid ligament. When this disposition of bone becomes very prominent the flat-foot enters on its permanent stage, and the more marked the projecting angle becomes, the greater is the resistance offered to the reduction of the deformity. The angle ultimately projects so much that it locks on the scaphoid, and no reduction is possible until it be removed.

The relaxation and subsequent alteration of shape in the bones of Chopart's joint are the key to the disease and its successful operative treatment.

There are two secondary changes in the foot, however, that deserve notice as completing the picture of flat-foot —first, the everted or valgus position, and second, the attitude of the great toe. It naturally follows that, after the descent of the arch on the inner side of the instep, the changed relations of the tarsal bones produce an appearance of the foot resting unnaturally on its inner edge, or in other words, becoming everted. In most cases of flat-foot this phenomenon is very apparent. It seems due to the falling down of the inner arch, and is generally unconnected with valgus ankle. When the arch is raised into its proper place the eversion vanishes.

By the increased separation of the extremities of the arch the structures of the sole of the foot are made tense, and the muscles that flex the great toe, being put on the strain, and even atrophied by the pressure against the ground, flex the great toe at its ball, and it consequently ceases to form an angle with the metatarsus, and comes to be in a straight line with it. In this way the metatarso-phalangeal joint appears very prominent above, and from its abnormal exposure to pressure is tender or forms a bunion, while the ball of the great toe, a feature of the normal sole, is diminished in size. The position of the toe is at first remediable, and a restoration of the arch of the instep produced at Chopart's joint by the hand remedies at the same time, without anything further being done, the malposition of the toe, but it reappears in proportion with the reappearance of the flattened arch. In long-standing cases the toe becomes permanently deformed, and may ultimately give rise to much suffering.

In the very worst cases, which are rare, the yielding of the foot at Chopart's joint goes even further than to allow the head of the astragalus to touch the ground. The sole divides into two parts, that before and that behind Chopart's joint, and each obeys a different tendency. The part anterior to the joint remains as the walking sole of the foot and continues in contact with the ground, while the other portion, consisting of the os calcis and astragalus, has its posterior end drawn up so that the tuberosity of the calcaneum is elevated, a finger's breadth or thereby, from the ground, and the portion of the sole corresponding with the posterior fourth of the foot does not come into contact with the ground. The bones are rigidly fixed in this perverse posture, and the feet are shaped, not like arches, but like canoes (Fig. 2).

The difference between a flat-foot where the deformity is still reducible and one where it has become rigid depends upon a change of form of the bones, already partly described, that can to some degree be observed by palpation from without, but which is very evident when the joint is opened.

In the extreme dorsal flexion of the astragalo-scaphoid joint present in flat-foot, the caput tali is no longer so much covered below by the scaphoid as it normally is, but escapes from it downwards and inwards, so that it finally forms on the inner side of the sole a prominence greatly exceeding in size that of the tuberosity of the scaphoid. The articular surface of the scaphoid is altered in direction, so that it looks more downwards, and tends to forsake its contact with the caput tali. There would be an actual gap between the bones (Fig. 3) below did not the astragalus accommodate itself to the void and assume an angular form with two facets nearly at right angles to each other, one looking forwards and articulating with the scaphoid, and the other looking downwards to the ground, parallel to it and resting on the inferior calcaneo-scaphoid ligament (Fig. 4).

So soon as the altered shape of the astragalus head becomes pronounced the deformity ceases to be easily reducible, and it shortly comes to pass that it offers an insuperable obstacle to the reduction; the foot is henceforth fixed, because the prominent angle cannot be made to ascend behind the scaphoid, since it locks upon it with every attempt at plantar flexion.

I have given much attention to these cases and have tried many plans of treatment. Boots with the inner margins of the soles raised, arched steel supports under the inner side of the sole, well moulded pads of cork and other materials, or hollow cushions of caoutchouc, have not produced a cure even in mild cases, while they are of course inapplicable in the severer forms. Lateral supports to counteract the valgus position have been equally unsuccessful. Prolonged rest, with or without stiff bandages, has always relieved the pain for the time being, but has never cured a single case in my hands. Neither have I had any success with Langenbeck's method of forcibly reducing the deformity with the hands, and then treating in plaster of Paris bandages, for it is inapplicable in the reducible cases and impossible in the more advanced forms. To give the experience of a good many years in one sentence—none of the plans of treatment I have tried have had in my hands any effect whatever in the cure of the disease.

When observing the disease and my failures in its treatment, I could not help being struck with the prominent part played in its production by Chopart's joint, and became convinced that, could any method be devised of restoring the joint to its normal position and rigidity, or even of causing bony ankylosis there, it would almost surely result in a cure. From observing that so long as the manipulating hand held Chopart's joint firmly reduced, so long the deformity was apparently cured, the inference was natural that any method of imitating from within the effect of this support from without would have a good prospect of radically curing the disease. Ankylosis between the astragalus and the scaphoid could do no great harm, for there are so many other points at which the tarsus is movable that probably the rigidity of this one joint would entail no inconvenience.

The first attempts at reducing this idea to practice were made in the year 1877, when a series of cases of flat-foot were treated by reducing the deformity as perfectly as possible, fixing them in the improved position by plaster of Paris bandages and maintaining them immovable for periods of three months. Some of them had a fenestra cut in the bandage and frequent injections of carbolic acid lotion made into the neighbourhood of the joint in the hope of causing rigidity by the prolonged rest and the irritation of the injections. Although some seemed to have benefited by the treatment the improvement was not permanent, and in none was a satisfactory cure obtained.

It was next decided to open the joint and remove the articular cartilage from a small portion of either bone at corresponding points, under the idea that ankylosis would result from it. Bidder's experiments, performed in 1877,* had shown that new bone is not formed when an ivory peg is driven through the articular surface of a bone, and hence it was inferred that nailing the bones together by pegs, without removal of the articular cartilage, would

* *Langenbeck's Archiv.*, vol. xxii., heft i. *Regeneration des Knockengewebes.*

fail in producing ankylosis, while removal of a portion of the articular cartilage would probably result in bony union of the denuded parts.

Accordingly in the next case that was submitted to treatment an incision was made along the inner side of the foot over the joint, and while the foot was held in the best possible position a notch was made by a small saw in the two bones, the saw being made to cut a linear track into the head of the astragalus and through the joint for some distance into the scaphoid (Fig. 5). It was hoped that bony union between the two clefts would result, and to favour this the foot, still held in the rectified position, was put up in plaster of Paris. After three months' rest this patient was dismissed much improved, although I am unable to state that her cure was permanent, as careful inquiry failed to trace her after she had left. Before she left I believed I could make out slight movement at Chopart's joint, and as this would have jeopardized the cure it was decided to treat the next cases by more extensive denudation.

In the year 1878 two patients were subjected to the following operation. An incision was made along the inner border of the foot down to the joint, and a small wedge of bone, three-quarters of an inch deep and of a like breadth, was chiselled out of each of the bones, leaving notches at points corresponding with each other, the foot being held in the position of most complete rectification while this was being done. In both cases the patients were dismissed in two months seemingly cured: in one of them the cure was permanent a year after the operation, in the second case the result was not satisfactory, as she was still complaining nearly two years after the operation.

Yet it seemed probable that a more certain means of producing bony ankylosis would yield better results, and, after much consideration, the following method was devised and has since been carried out with excellent results in seventeen cases.

The object of the proceeding is to denude as much of the cartilaginous surfaces of the astragalo-scaphoid joint as can conveniently be reached, to place the foot in proper position and secure its immobility by uniting the two bones by ivory pegs. It is applicable both to the milder cases which are reducible, and the severer, which are not so. I have never had an opportunity of using it in the very aggravated forms where the sole becomes boat-shaped and the heel leaves the ground.

On purpose to secure the utmost benefits of the antiseptic method, the feet are, as a preliminary step, washed daily in a 1.20 carbolic lotion and done up in a large Lister's dressing, while the patient is confined to bed, and this is carried out for four or five days, so as to secure the greatest possible purity of the thick layers of epidermis that exist on the sole and over Chopart's joint. All loose epidermis is peeled or rubbed off with pumice-stone, and when a state of purity has been reached the patient is put under chloroform or ether, both feet, if the disease be double, being operated on at one sitting.

The dressings are removed, the elastic tourniquet is placed round the leg below the knee, and the leg, ankle, and foot are washed once more with carbolic lotion and finally with oil of turpentine. The foot is then laid upon a disinfected piece of macintosh and the carbolic spray is turned on it. It is convenient to stand on the left side of the patient, while an assistant stands opposite, holding the foot by the ankle and metatarsus.

In a normal foot Chopart's joint lies about an inch in front of the internal malleolus, and the most prominent bony point on the inner side of the foot is the scaphoid tuberosity just anterior to it. But in the flat-footed the astragalus head is so greatly displaced from the scaphoid and so prominent that it forms the large projection seen and felt on the inner side of the foot about an inch in front of the tibia, and the scaphoid is comparatively indistinct, while the line of Chopart's joint is half an inch further from the tibia than usual. By moving the metatarsus this can generally be felt to be the case. Hence an incision, to open the joint, has to be made further from the ankle than in a normal foot.

The foot, lying in the assistant's grasp without any attempt being made to reduce the deformity, is placed with its outer side resting on the operation table, and an incision, an inch and a quarter long and parallel to the sole, is made along its inner side over the joint, dividing all the structures down to the bones. If this incision commence about an inch from the tibia its centre will be over the articulation. No important structure is divided save some small branches of the internal saphena vein or the small vein itself, and these may be tied with catgut or left unligatured.* If the incision be carried down to the bones by the first movement of the knife the head of the astragalus, partly covered with cartilage, will be observed through a button hole in the capsular ligament in

* A longitudinal incision gives best access to the joint: it may, in aggravated cases, advantageously be slightly curved with its convexity downwards, if it seem desirable. An incision across the border of the foot, parallel to the line of articulation, is attended with more risk of missing the joint and wounding important structures, while it gives less ready access to the head of the astragalus than the incision parallel to the sole.

the depth of the wound if its edges be retracted by aneurism needles. If not, a second cut with the knife completes the division of the soft parts. After the caput tali has become visible free access to the joint has to be obtained by separating the attachments of the ligamentous capsule to the edge of the scaphoid for a distance of half an inch on each side of the wound. The ligament is seized by a dissection forceps, elevated, and detached from its insertion into the scaphoid, its connections with the periosteum and fibrous structures over the scaphoid being maintained as far as possible by cutting with the edge of the scalpel directed towards the toes, the blade lying parallel with the bone.

In this manner a somewhat T-shaped opening is made into the joint, and sufficient access is gained for the next step of the operation—the denudation of the bones. A stout chisel, half an inch broad, bevelled on one side and provided with a wooden handle, is held in the right hand with the bevelled side away from the caput tali, while by its means the articular cartilage is shaved away from the whole of the exposed surface of the bone over as great an extent as possible, a thin layer of the subcartilaginous bone being also removed, so that the cancellous structure is well laid bare. The chisel is next applied to the scaphoid, the bevelled side being held towards it, as the surface to be here denuded is concave, and by repeated shavings the denudation is carried as far as possible between the bones. In this manner each bone is bared of its cartilage, and if the arch be now restored to its normal position by the assistant the two surfaces are found to correspond, the head of the astragalus retreating into its normal position behind the scaphoid.

If the deformity be of old standing and the bones have adapted themselves to their altered position, it is not possible to restore the arch until, by means of the chisel, the prominent angle that has formed on the lower surface of the astragalus head has been shaved off and the rounded form of the head restored: but when this is accomplished the arch is easily put into its proper shape.*

The next step of the operation is to nail the bones together by ivory pegs. The joint is washed out with 1.20 carbolic lotion, and the arch of the foot is restored to its normal shape by the assistant depressing the metatarsus. The scaphoid after this movement covers the head of the astragalus and the denuded surfaces lie opposite one another, separated by an interval of about a quarter of an inch, caused by the removal of so much bone. In the milder cases the arch is perfectly restored to its position, even in the severer cases it is much improved. In its restored shape it is held fast by the assistant, while the operator drills two holes through the scaphoid into the caput tali. The anterior angle of the wound is drawn apart by retractors so as to expose the scaphoid. The point of the drill† is placed on the upper and inner side of the scaphoid, the drill is pointed towards the centre of the caput tali, and when its direction has been well determined it is set in motion, piercing a

* The denudation can be accomplished by means of a stout scalpel, a Volkmann's sharp spoon or curette, or a mallet and chisel, but the most convenient plan has seemed to me that which I have described, by the chisel held in the hand. The soft bones are easily denuded, and the shavings are carefully lifted out by a dissection forceps. A little gluey synovia often escapes during the process.

† The best drill, to my thinking, is an Archimedean drill, such as is used by carpenters, fitted with what are sold as "broaches" (No. 40) by the wholesale watchmakers, and which form good drill points. They should be somewhat less in size than the ivory pegs to be employed. The drills cost 3/-, the broaches 3½d. each.

hole an inch and a quarter in depth through the two bones. On withdrawing the drill* an ivory peg† is held ready to be put into its place. When it is withdrawn one of the pegs is placed in the hole and driven home by a series of light taps with a small mallet. After it has entered for rather more than half its length its projecting end is cut off level with the scaphoid by a bone forceps. A second perforation is then made parallel to the first and nearly half an inch distant from it through both bones, and a second peg is driven home in it. One of the perforations may be made beneath one margin of the skin and the other beneath the opposite margin (Fig. 6).

The bones are so firmly fixed together by the pegs, which can be seen crossing the gap that separates them, that when the assistant removes his hand, the foot remains in the improved position.

The wound is well cleaned, its lips brought together by a series of catgut sutures an eighth of an inch apart. I do not usually loosen the elastic tourniquet until after this has been done and the Lister's dressings applied, but if there be any dread of hæmorrhage the elastic cord can be removed before the wound is closed. If this be done the bleeding is usually free, and comes from the bones.

* The best method of disinfecting the drill and its point is, I believe, to wash it well with oil of turpentine.

† The pegs are prepared from the finest ivory knitting needles, sold in the Berlin wool shops as ivory knitting pins, No. 13, Wynn's bell gauge, and are about eight inches long and of the size of a No. 7 or 8 catheter, French scale, or a No. 2 English scale, They should be cut with a fine saw into pieces three inches in length, which are sharpened at one end by a file, and boiled in 1.20 carbolic water for ten or fifteen minutes until they lose their whiteness and become impregnated with the disinfectant lotion· after which they are preserved in 1.8 carbolic oil. Or they may be prepared by Neuber's method of prolonged soaking in German oil of juniper and afterwards preserved in absolute alcohol. I have not yet used them as prepared by the latter method.

But as no arteries of any magnitude have been divided the compression of a well applied dressing is the best means of preventing the escape of any quantity of blood. A Lister's dressing is therefore carefully put on, enclosing the whole foot from a hand's breadth above the ankle to beyond the toes. The spray is then turned off. A few turns of plaster of Paris bandage outside the dressing steady the foot, and after they have been applied the tourniquet is removed.

The patient suffers sharp pain for four and twenty hours, and after that the convalescence is painless. The dressings may be removed on the fourth day, or left on for weeks. On their removal the wound has always been found healed, or existing merely as a superficial sore. The patients are kept in bed for two or three months, and are then permitted to rise. In a week or two afterwards they are able to walk freely.

This operation I have performed seventeen times in ten patients, and in one case, operated on the 8th December last, I pegged on the left foot the joint between the scaphoid and internal cuneiform as well as Chopart's joint, because its movement seemed unusually free.

My patients have all, without exception, remained free from fever, the thermometer not rising above 100° Fh., save when two of them, while still under treatment, were attacked by sore throat, lasting for a day or two. The wounds remained aseptic, and the dressings were renewed only a very few times. In some of the patients a slight tenderness and pain on trying to walk at the end of six or eight weeks indicated that the joint was still unankylosed, doubtless from the slow repair due to defective circulation. They were often permitted to walk a little at the end of two months, and to use the feet freely at the end of three months, but I think that any use of the feet should as a rule not be permitted until three months have elapsed, as bony union is slow in such individuals.

In all my patients, to the best of my belief, great benefit resulted from the operation, and in most of them bony ankylosis and a painless arch was obtained. They mostly resumed their laborious occupations, and were able fully to bear all the demands made upon them. One patient subsequently died of heart disease.

In one patient an ivory peg was spontaneously and painlessly extruded by a small cutaneous opening five months after the operation; but in every other instance they remained, probably to undergo slow absorption or vascularisation.*

Another patient, while under treatment, complained that one of his operated feet ached a little, and was never quite satisfied with the result on that foot. Yet he underwent a season of heavy harvest work after leaving the Infirmary. He returned again last November, and a little fulness was then detected about the calcaneo-cuboid joint of the right foot, the one which had ached. Both were put up in plaster of paris for two months, and when these were removed at the beginning of this month the feet looked perfect. The restoration of the arch of the instep was absolutely perfect in this case.

In a few of the other patients the plantar arch was restored to perfection. But this was not generally the case. In all it was, however, much improved, and added to the patients' lightness of step even when, to a surgical eye, traces of the deformity remained.

* *Riedinger. Verhanp. d. Deutschen Gesell. f. Chir.*, 1881, x. Cong., p. 167; *Trendelenburg*, the same, 136.

Fig. 1.

Valgus Ankle.

Fig. 2.

Extreme flat Foot.

Plate 11.

Fig. 3.

Diagram of the tendency to separation of the Bones in flat Foot.

---

Fig. 4.

Diagram representing the sort of alteration that occurs in the shape of the Caput Tali. It is not possible to represent the change diagrammatically, as the Caput Tali slips from behind the Scaphoid downwards and inwards, not merely downwards as shewn in Fig. 3.

Fig. 5.

An early attempt at cure by Operation.

a. Line of saw incision.

b. Depth of saw incision.

Fig. 6.

Sketch of the Operation by pegging Chopart's Joint.

One of my cases is still under treatment.

Knowing how much the prolonged rest following the operation would of itself tend to improve such cases, and alive to the temptation to be sanguine and over confident in judging of the results of one's own work, I have endeavoured to free myself from any bias, and to form a true estimate of what was due to the operation itself in the improvement that resulted. Such of the patients as were accessible were seen or written to at considerable periods after the operation, and were warned that the information they gave was required not to gratify me, but to determine whether the same operation should be tried upon others. They all, save the patient mentioned above, maintained that the operation had cured them, and that they would willingly undergo it again on account of the benefit they had derived from it.

My own impression is that this method of treatment is likely to be of use in suitable cases, uncomplicated by other diseases, and where none of our other methods can be relied upon. I therefore think it desirable that it should be tested by others, should they deem it worthy of a trial, and receive its verdict according to the results it may yield in their hands.

### TABLE OF CASES OF FLAT-FOOT OPERATED BY EXCISION AND PEGGING OF THE ASTRAGALO-SCAPHOID ARTICULATION.

| No. | Sex. | Age. | Occupation. | Right or Left Foot. | Date of Operation. | Course of Cure. | Date of Dismissal. | Final Result. |
|---|---|---|---|---|---|---|---|---|
| 1 | F | 18 | Farm Servant... ... | Right and Left | 4th Jan., 1880 | Recovery afebrile save for intercurrent sore throat | 5th June, 1880 | Apparently cured at dismissal. Writes on 10th Dec., 1883, that she is "quite well, much better of the operation," her feet "stronger than they were before." |
| 2 | F | 14 | Domestic Servant... | Right ... ... | 30th Sept., 1880 | Afebrile recovery ... | 22nd Nov., 1880 | Sent to Convalescent Hospital, and subsequently dismissed cured. On 1st April, 1881, returned to report herself. Had been in service for a month and was nearly well. On 8th Sept., 1881, she returned to report and was seen by Mr. Wm. Adams, London. Quite cured. |
| 3 | F | 17 | Farm Servant... ... | Right and Left | 30th Sept., 1880 | Afebrile recovery ... | ? | Returned to report herself on 29th Sept., 1881. Feet almost perfect in shape, valgus position gone. Is satisfied she is cured. |
| 4 | F | 13 | Mill Worker ... ... | Right and Left | 5th Mar., 1881 | On 24th May, 1881, pain on walking. Again kept at rest | 7th July, 1881 | Apparently cured at dismissal. She died, I was afterwards informed, of heart disease and general dropsy in June, 1882. |
| 5 | F | 15 | Crofter's Daughter | Left ... ... ... | 25th Jan., 1882 | Afebrile recovery ... | 11th Mar., 1882 | Apparently cured at dismissal. Writes on the 8th Dec., 1883, that the operation improved her foot very much, though it is "weak at times yet." |
| 6 | F | 17 | Domestic Servant... | Left ... ... ... | 25th Jan., 1882 | Afebrile recovery ... | 13th Mar., 1882 | Returned to report herself 9th Oct., 1882. Chopart's joint seems ankylosed at inner side. Patient is satisfied she is cured. |
| 7 | M | 18 | Farm Servant... ... | Right and Left | 30th Dec., 1882 | Afebrile recovery ... | ? | Complained of a little pain in right foot on dismissal. Supposed to be malingering. Worked through harvest of 1883 and returned on 1st Nov., 1883. Outer part of right Chopart's joint a little swelled. Left foot perfect. Put up in plaster bandages until 5th Jan., 1884. The feet were then in perfect shape and seemed soundly cured. |
| 8 | F | 18 | Domestic Servant... | Right and Left | 1st Mar., 1883 | Afebrile recovery ... | ? | Returned 22nd Oct., 1883, and reported herself cured. Both feet perfect. A month previously an ivory peg painlessly appeared at a small opening and was drawn out. |
| 9 | M | 14 | School Boy ... ... | Right and Left | 9th Oct., 1883 | Afebrile recovery ... | 27th Nov., 1883 | Sent home with every appearance of being perfectly cured. |
| 10 | F | 12 | Farm Servant... ... | Right and Left | 8th Dec., 1883 | Afebrile recovery ... | ... ... ... ... | Still under treatment: giving every promise of a good result. |

## Figures and Tables

**Fig. 1. f1:**
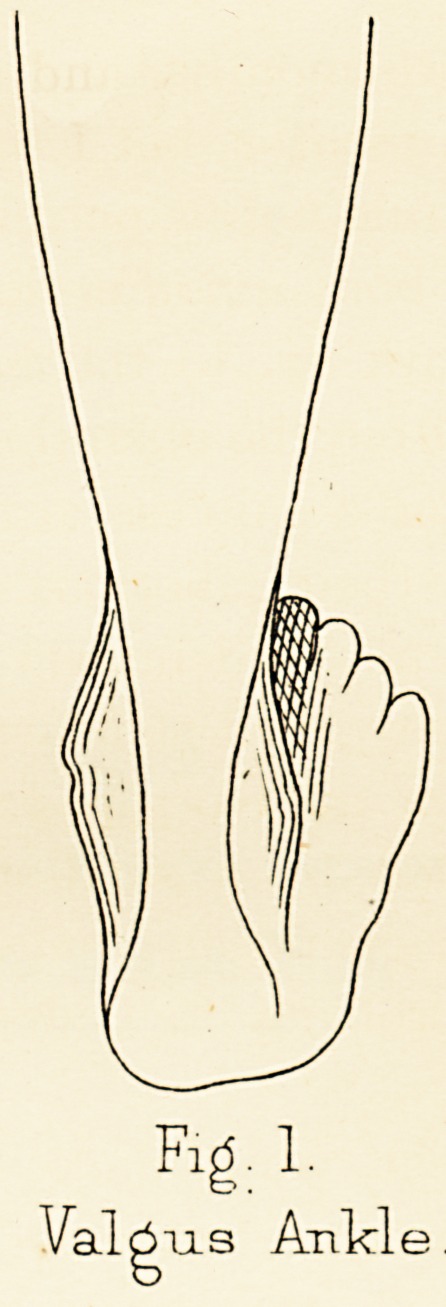


**Fig. 2. f2:**
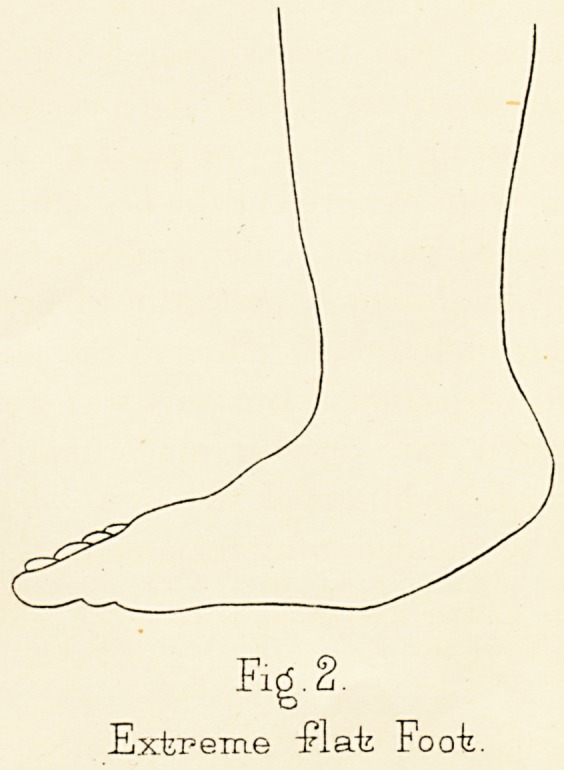


**Fig. 3. f3:**
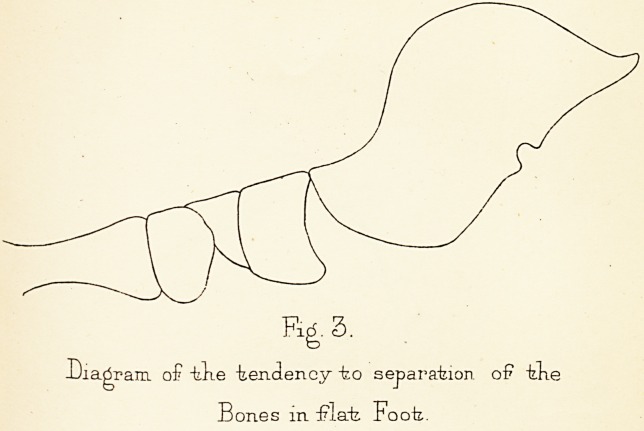


**Fig. 4. f4:**
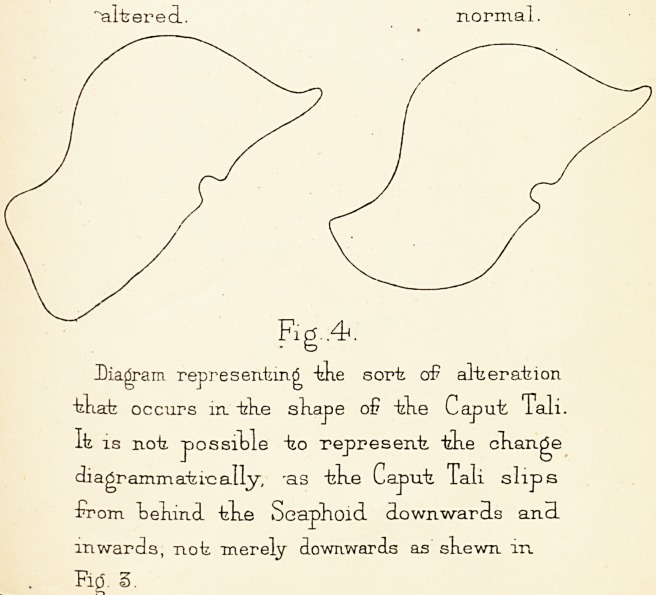


**Fig. 5. f5:**
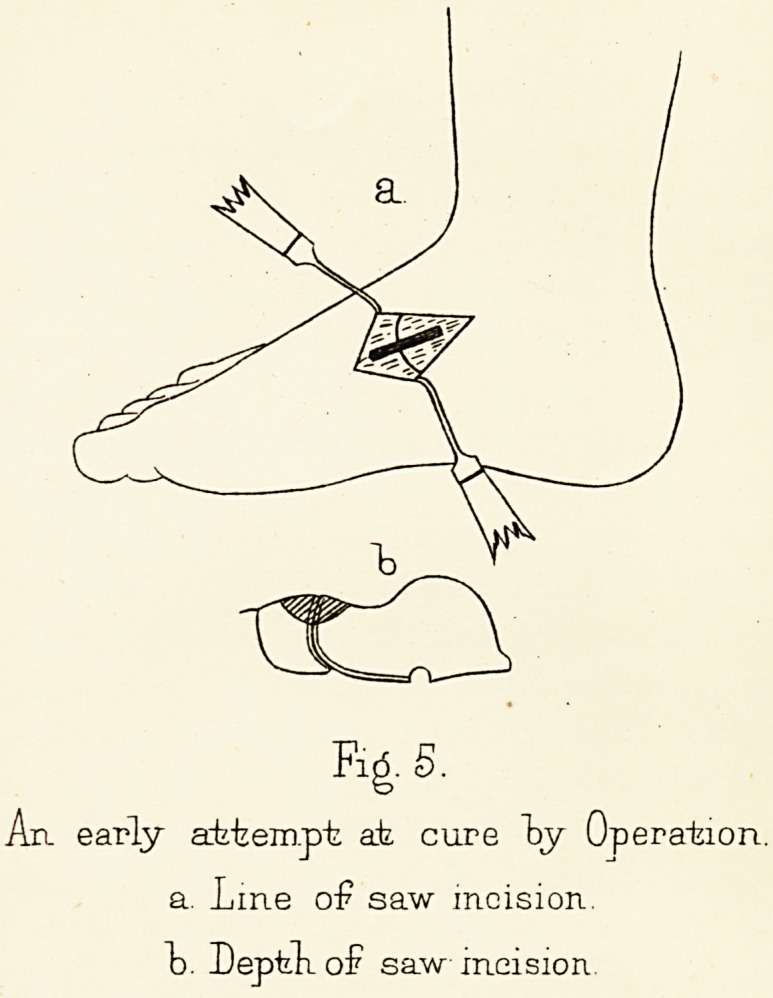


**Fig. 6. f6:**